# The secretion and retraction processes of pollination drops of ancient *Platycladus orientalis* and their response to different pollen types

**DOI:** 10.3389/fpls.2025.1669052

**Published:** 2025-10-29

**Authors:** Wei Zhou, Liyan Gong, Xiaobing Wang, Nawen Li, Xin Zhang, Jianjun Liu

**Affiliations:** ^1^ School of Art and Design, Guilin University of Electronic Technology, Guilin, Guangxi, China; ^2^ School of Life Science and Technology, Xinxiang University, Xinxiang, Henan, China; ^3^ Key Laboratory of Silviculture on the Loess Plateau State Forestry Administration, College of Forestry, Northwest A&F University, Yangling, Shaanxi, China

**Keywords:** pollination drop, *Platycladus orientalis*, pollen, ancient gymnosperm, secretion, retraction

## Abstract

**Introduction:**

The pollination drop (PD) is an important ovular secretion that occurs during the pollination stage in gymnosperms.

**Methods:**

In this study, ancient *Platycladus orientalis* trees near the Mausoleum of the Yellow Emperor were selected to investigate the secretion and retraction processes of PDs and the response of PDs to different pollen types.

**Results:**

In a natural setting, PDs were secreted in the early morning and retracted around noon, with the appearance time extending equally to both sides of 6 a.m., and there was a daily circadian rhythm of the variations. Similar changes were observed under *in vitro* conditions. Immediately after pollination, the PDs were retracted rapidly for a short time, after which the retraction rate gradually decreased for the next 20 min, and then remained almost unchanged, suggesting that there may be a comprehensive retraction mechanism for PDs. After pollination, the PD retraction rate decreased with decreasing pollen viability and botanical affinity, suggesting the presence of substances used for pollen identification, an identification mechanism and the presence of specific forms of identification or communication with pollen. The ability of the PDs of ancient *P. orientalis* trees to identify and respond to pollen increased with increasing tree age.

**Discussion:**

The secretion and retraction of PDs may be two mutually exclusive processes that cannot be considered together. The younger the tree was, the stronger the driving force of secretion, as demonstrated by the higher PD secretion rate, larger surface area, and greater duration. The older the tree was, the stronger the driving force of retraction, as demonstrated by the significantly higher retraction rate of PDs after pollination. Each process has its own advantages and disadvantages in terms of pollination efficiency, but the decrease in the PD secretion rate by ancient *P. orientalis* was significantly lower than the increase in the PD retraction rate. In summary, female cones of older *P. orientalis* exhibit a greater reproductive ability than those of younger *P. orientalis*.

## 1 Introduction

*Platycladus orientalis* (L.) Franco is the only species of the genus *Platycladus* in the Cupressaceae family. *P. orientalis* is native to North China and is an adaptable evergreen gymnosperm tree species with a long cultivation history. China is rich in ancient *P. orientalis* resources. These ancient *P. orientalis* trees are a large and valuable gene bank, a germplasm resource bank, a living specimen for the study of historical and environmental changes, and have important and unique scientific and humanistic value (Yang, 2014; Zhou et al., 2019). However, the future of ancient *P. orientalis* trees based on their current protection is not optimistic. While strengthening the protection of ancient *P. orientalis* trees, it is highly important to explore their reproductive functions to better understand these trees and thus support their continuation, preservation and rejuvenation.

The pollination drop (PD) is a liquid secretion produced by the female cones of most gymnosperms during pollination, and is a key part of successful pollination. Pollen adheres to sticky PDs and is pulled into the pollen chamber at the apex of the nucellus as the PD is retracted (Wang et al., 2015), which increases the contact area between the micropyle and air (Wang et al., 2010); this process thus guides pollen growth (Shimizu and Okada, 2000) and filters pollen pollution (Runions and Owens, 1996; Tomlinson et al., 1997). This pollination mechanism greatly improves the pollination efficiency of gymnosperms (Owens et al., 1980; Tomlinson, 1994; Anderson and Owens, 2000; Mugnaini et al., 2007; Bringas et al., 2025). The PD was reported as early as 1871 (Strasburger, 1871). Subsequently, Tison (Tison, 1911) photographed PDs of *Cupressus funebris*, which contained airborne pollen, for the first time. With the advancement of technology and in-depth studies, the formation and secretion patterns of PDs have become increasingly clear. The phenomenon and pattern of PD secretion by different plants during the pollination period have been reported (McWilliam, 1958; Owens et al., 1987; Tomlinson et al., 1997; Anderson and Owens, 1999; Xing et al., 1999; Anderson and Owens, 2000; Jin et al., 2012a, 2012; Dörken and Jagel, 2014; Breygina et al., 2024; Deng et al., 2025). However, very little is known about the pattern of PDs of *P. orientalis* during pollination; Xing et al. (Xing et al., 1999) conducted a preliminary exploration, but many details are still unclear. In addition, there are no reports on the secretion and retraction patterns of PDs of *P. orientalis* of different ages. Elucidation of the secretion and retraction patterns and intrinsic relationships of PDs of *P. orientalis* of different ages can provide important reference information about the function and mechanism of PDs of *P. orientalis* and can help to further improve the pollination efficiency of *P. orientalis*; thus, this information has a great practical significance.

The main factors affecting the capture of pollen by PDs are the persistence time and the surface area. The longer the persistence time and the larger the surface area are, the greater the probability of the PD contacting pollen, and the greater the likelihood that pollination can be completed. Therefore, these are the two important factors affecting the pollination efficiency of PDs. In this study, an observation method was used to investigate the secretion and retraction processes and characteristics of PDs of *P. orientalis* under different pollinated and unpollinated states with the aim of identifying the patterns of PDs and their relationship with tree age.

## 2 Materials and methods

### 2.1 Plant material collection

**Figure 1:**
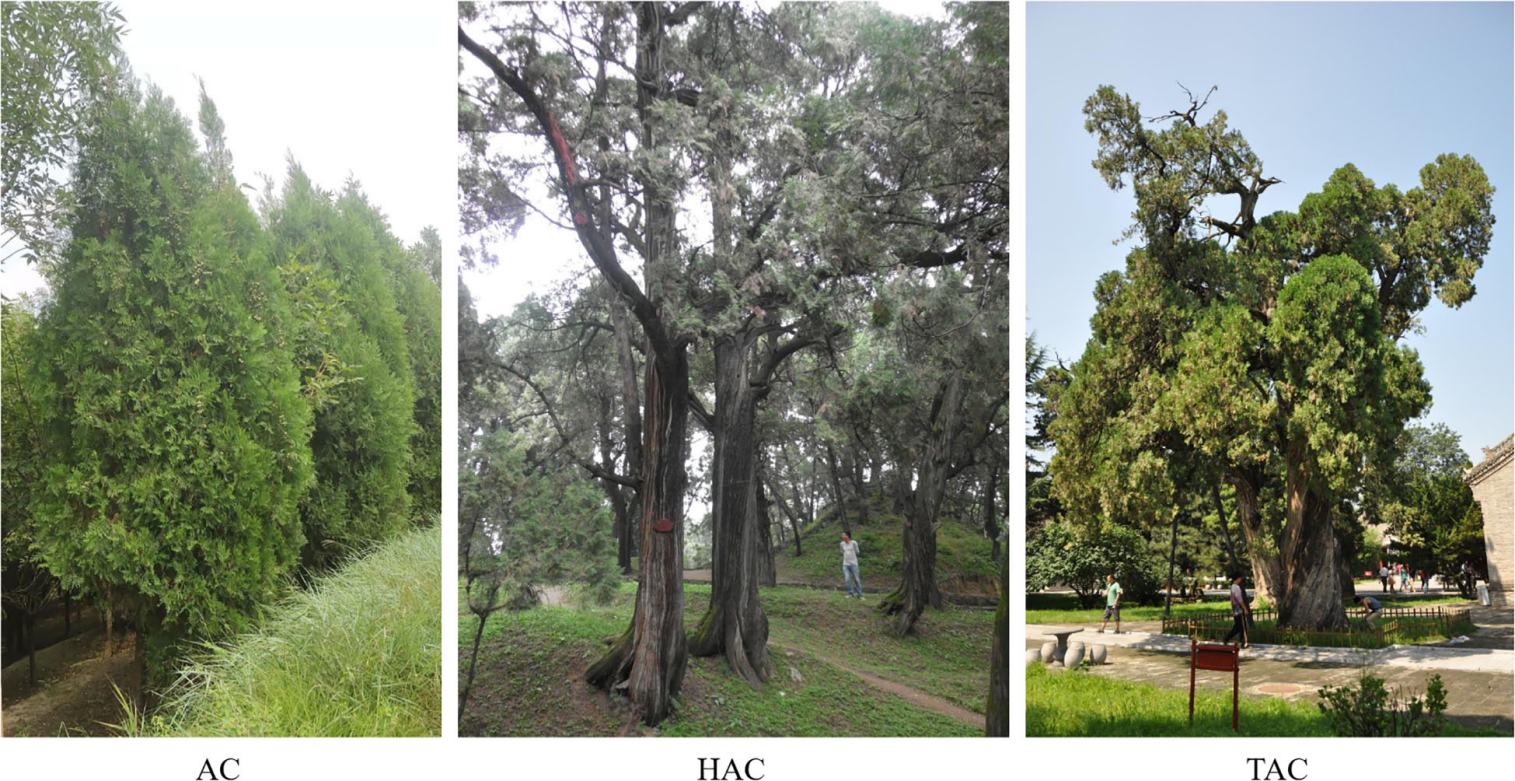
Three age sample gymnosperm photos.

In this study, two ancient *P. orientalis* age groups were used, namely, thousand-year ancient cypress (TAC) and hundred-year ancient cypress (HAC), and the control group was referred to as adult cypress (AC). The TAC group includes 3000-year-old cypress trees with a diameter of approximately 1.8 m, the HAC group includes 500-year-old cypress trees with a diameter of approximately 70 cm, and the AC includes 30-year-old cypress trees with a diameter of approximately 20 cm. Tree age was determined based on the archives of the Mausoleum of the Yellow Emperor. There were significant differences in tree volume and age between the groups (**Figure 1**).

**Figure 2:**
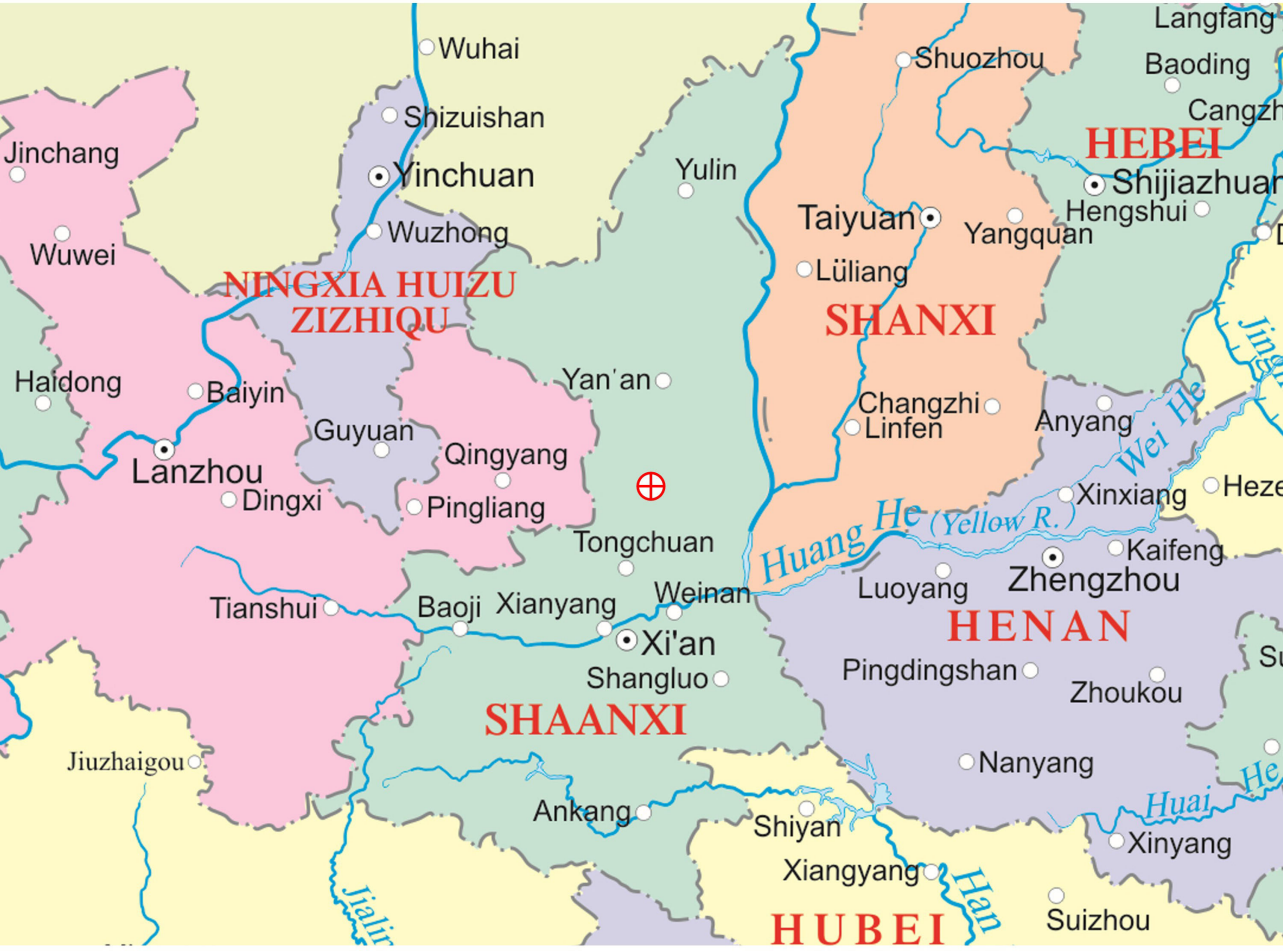
Location map of sample trees. The red cross in a circle represents the position of sample trees.

Based on the Mausoleum of the Yellow Emperor of Huangling County (35°34′58.3″N, 109°15′34.7″E), Yan’an city, Shaanxi Province, P. R. China, three healthy and representative trees belonging to the AC, HAC and TAC groups were selected from similar environments, as three independent biological replicates. The trees were labelled, and their locations were recorded (**Figure 2**). On March 2, 2015, when the bract scales of the female cones were about to open but had not yet opened, small branches with female cones were collected from each sample tree, and at least 50 female cones were collected for each group. The cones were emasculated, brought indoors to prevent natural pollination, placed into a 10 mL triangular flask containing water, and labelled. Five female cones from each group were used for the indoor PD secretion/retraction tests in the unpollinated state, and the rest were used for the tests in the pollination state.

### 2.2 Major instruments and reagents

#### 2.2.1 Major instruments and tools

The experiment involved the use of single-lens reflex (SLR) cameras (EOS 5D Mark II and EOS 60D, Canon, Japan), a microscope (BX43, Olympus, Japan), a stereomicroscope (EZ4HD, Leica Microsystems, Germany), a stereoscopic imaging system (Tucsen 5.0Mega Pixel CMOS Digital Microscope Camera, Tucsen Photonics Co. Ltd., Fujian, China), an artificial climate box, a sieve-like container, a headlamp, a magnifying glass, tweezers, scissors, pruning shears, a marker, a kraft paper bag, a rubber band, string, a GPS, batteries, labels, a vial, a glass rod, a small beaker (5 mL), a triangular flask, a sponge, a strand of hair, a goose feather earpick, rubber gloves, medical cotton, a mask, a nylon sieve (mesh), sulphite paper, aluminum foil, etc.

#### 2.2.2 Major reagents

Alcohol, silica gel, sucrose, boric acid, peptone, agar, etc. were the major reagents used in this work.

### 2.3 Experimental methods and procedures

#### 2.3.1 Observation of the secretion and retraction of the PDs of ancient *P. orientalis* in the unpollinated state

When the bract scales of the collected female cones were unfolded and PDs were produced, the morphology and the secretion and retraction processes of PDs in each age group under the unpollinated state were observed using a stereomicroscope (EZ4HD, Leica Microsystems, Germany), and the time of production and retraction was recorded. First, a dedicated imaging system (TCA-5.0C PACK, Tucsen Photonics Co. Ltd., China) and the stereoscope were connected to computer so that the video could be transferred from the eyepiece to the computer monitor. This allowed digital image acquisition to be performed during the observation period. Then, according to the secretion and retraction rates of the PDs, TCA-5.0C was used to take pictures of each tested cone every 20 min (when taking photographs, the magnification at the front lens elements was adjusted to 5.0, so that the scale of the object and the image was 1:100); the images were saved and processed by TSView 7.3.1.7 and ISCapture 3.5, the time was recorded, and the entire change process of the PDs was recorded by the images. Finally, the PDs of the female cones were measured and statistically analyzed to quantify the changes the PDs underwent. For each group of twigs, five female cones were selected as replicates.

#### 2.3.2 Observation of responses of PDs to pollen with different levels of viability

Male cones of ACs were collected near the Mausoleum of the Yellow Emperor. The collected male cones were packed in perforated sulphite paper bags, transported to the laboratory, and placed on sulphite paper in a sieve-shaped container. Silica gel was then added the container to keep the cones dry. After the pollen sacs cracked, the pollen scattered, and the scattered pollen was collected using a 200 mesh sieve, placed in a sterile sealed vial, mixed evenly, and labelled for future use. During the storage process, the temperature and relative humidity were kept as low as possible because low temperature and low humidity can prolong the storage period of pollen. *P. orientalis* pollen was collected in a 5 mL beaker, subjected to high-temperature treatments, such as burning and baking, for inactivation (as indicated by a change in color), and subsequently collected in a labelled vial. The pollen germination solution was prepared, and an *in vitro* germination test was conducted on treated and untreated pollen samples to ensure that the pollen viability was adequate. The measurement methods used were the same as those described in Section 2.1.3.3, and there were 3 replicates for each condition (treated and untreated pollen samples).

The twigs with female cones from each group were carefully inserted into Petri dishes with sponges moistened with water, and the dishes were labelled. After the PDs appeared and their size stabilized, PDs of the same size in each group were selected as the experimental focus. Clean hair that had been wiped with alcohol was successively dipped into *P. orientalis* pollen samples wit with different levels of viability, and PDs of each group were pollinated under a stereomicroscope (approximately 20 grains/drop). Photographs were taken before and immediately after each pollination effort (using specific photography methods and magnification levels), and the pollination time was recorded. The pollinated PDs were carefully set aside without disturbing the drops. The response and retraction process of each group of PDs to the pollen samples were repeatedly observed without interruption, photos were taken, and the time was recorded. Three PDs from each age group were selected as replicates. To avoid disturbance, the pollination experiments were performed in a separate room.

During the PD observation experiments, the indoor temperature was maintained at 18-20°C, and the humidity was maintained at 50%-70%.

#### 2.3.3 Observation of the responses of PDs to pollen from different species

Pollen from *Juniperus formosana* and *Pinus bungeana* that had been collected in the previous year and well preserved was obtained from the laboratory, placed in sterile sealed vials, and labelled for future use. Since March is the flowering period of *Magnolia denudata*, the stamens of healthy *M. denudata* were collected and placed on sulphite paper in a sieve, and silica gel was added to the container to keep the samples dry. When the pollen was completely dry and dispersed, it was collected and sieved through a 200 mesh sieve, placed in sterile sealed vials, and labelled for future use. The three types of pollen were used to pollinate the PDs of *P. orientalis* in each age group, and the pollen consumption of *J. formosana*, *P. bungeana* and *M. denudata* is approximately 20, 10 and 15 grains/drop, respectively. The experimental procedure was the same as that described in Section 2.3.2.

#### 2.3.4 Observation of the secretion and retraction of PDs of *P. orientalis* in the natural state

Among the ACs, HACs and TACs near the Mausoleum of the Yellow Emperor, three representative and healthy individual *P. orientalis* trees in each age group were selected as replicates; their locations were recorded, and the trees were labelled. Beginning with the appearance of PDs, the morphology and the secretion and retraction processes of PDs of unpollinated female cones on the sample trees in their natural setting were observed with a magnifying glass. The times of secretion and complete retraction were recorded, and the PDs were photographed with an SLR camera (EOS 5D Mark II and EOS 60D, Canon, Japan) every 30 min. For each sample tree, the complete process was observed for one cone. To prevent interference from pollen, the female cones were carefully bagged between observations and photographs, and male cones were removed from the bags.

In the early morning of March 20, another 2 female cones were selected from each sample tree to perform artificial pollination in the natural setting using hand pollination. The goose feather earpicks were used to gather a small amount of *P. orientalis* pollen and tapped twice above the target female cone to ensure that pollen fell on the PD. The retraction of the PDs was observed and photographed every 15 min until the PDs were completely retracted.

#### 2.3.5 Data processing

The horizontal diameter (parallel to the micropyle) and vertical diameter (perpendicular to the micropyle) of the target PD in each photo were measured with a ruler (Ruler 1.16), and the true lengths were obtained by converting the values based on the scale bar. The conversion formula used is D=d×r/100, where D represents the actual value of the PD, d represents the measured value, 100 is the length of the scale bar, and r is the conversion coefficient based on the pixel resolution; in this experiment, r was 2/3. All the measurements were completed on the same monitor to ensure that the resolution remained consistent. Each group of PDs was measured individually, and the variation in the size of each measured PD at each time point was determined.

Since PDs are ellipsoidal, a method for performing ellipsoidal calculations was used to calculate the surface area. However, because this method involves elliptic integrals, there is no analytical expression for direct calculation, and the approximate formulas given by mathematicians are either very complicated or not very accurate (Kumar and Vishnu, 2004; Ji and Bian, 2008; Guo et al., 2017). Therefore, in this study, the professional software Mathematica 11.3 was used for automatic calculation and analysis of the PD surface area.

Excel 2013 was used for preliminary data processing, and SPSS Statistics 23.0 was used for statistical analysis. The secretion and retraction processes of the PDs in each age group under different states (pollinated and unpollinated) were analyzed separately to allow the patterns to be summarized.

## 3 Results and analysis

### 3.1 The secretion and retraction processes of nonpollinated *P. orientalis* PDs

**Figure 3:**
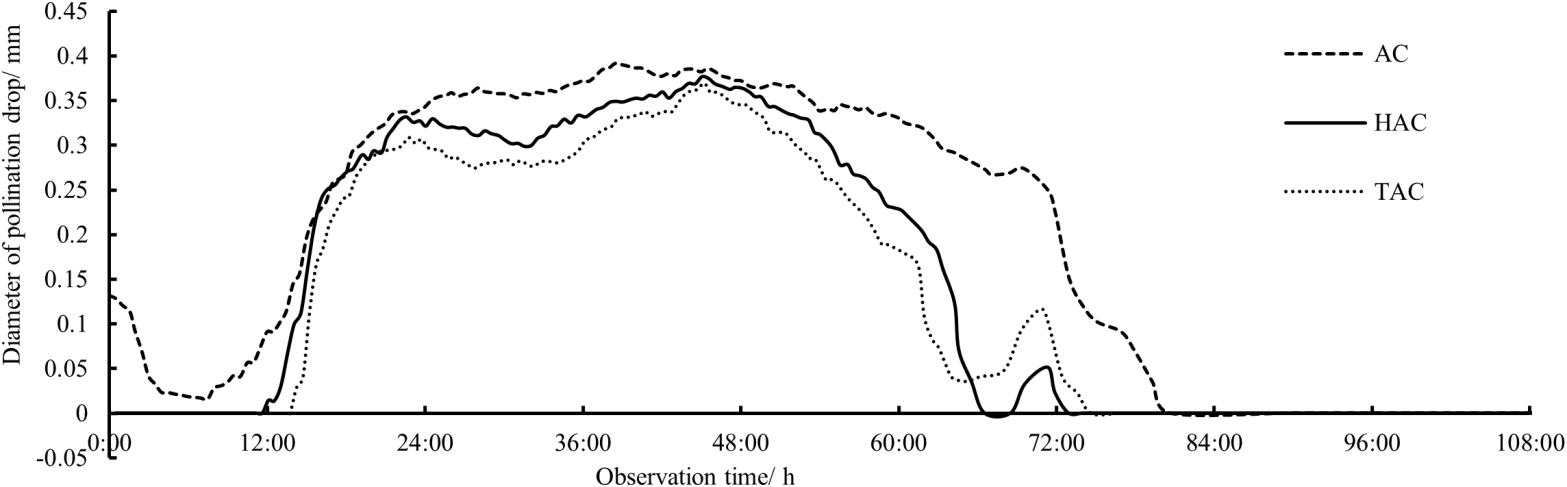
Processes of size changes of unpollinated PDs at different ages of *P. orientalis.*.

**Figure 4:**
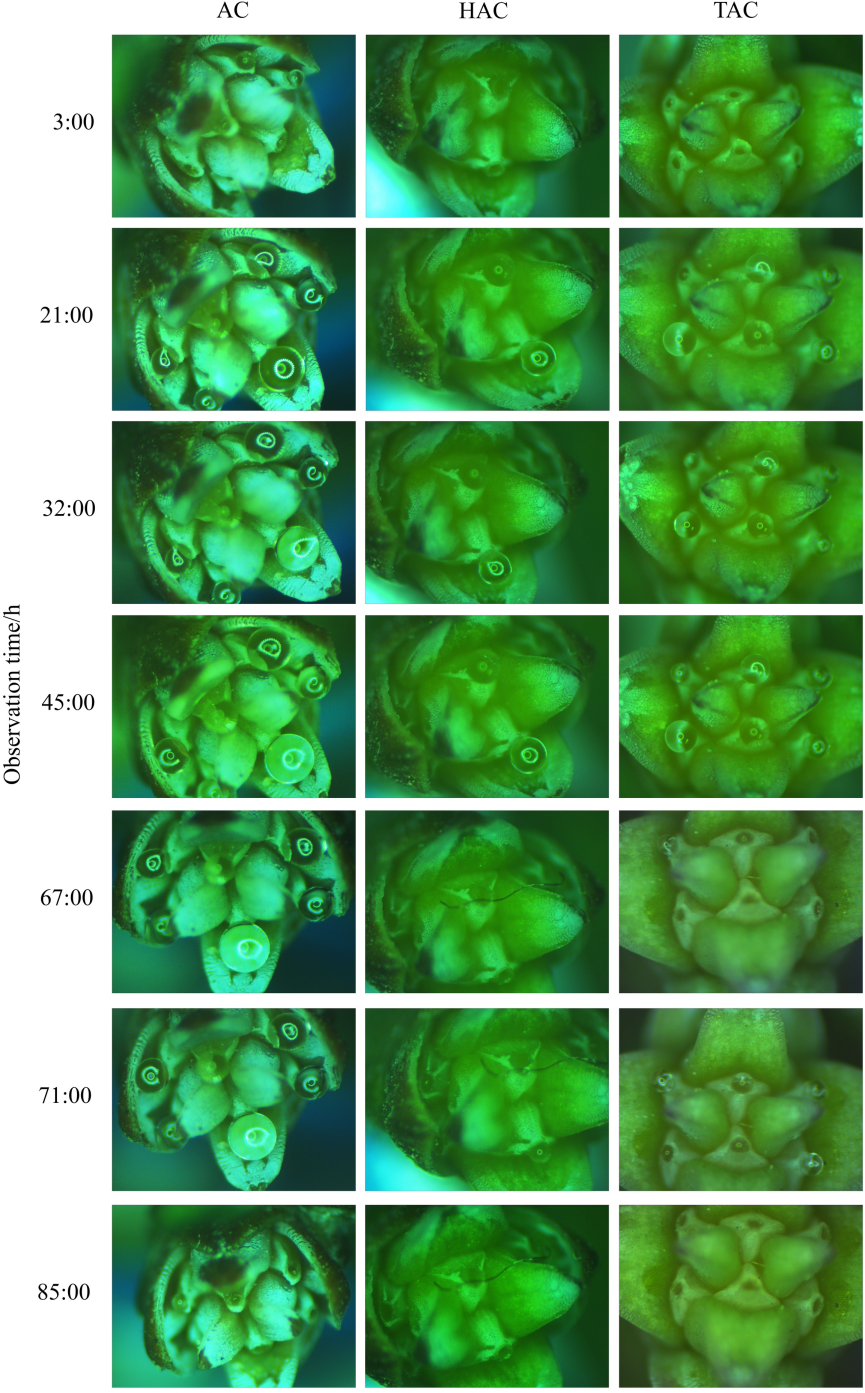
Process of secretion and retraction of unpollinated PDs at different ages of *P. orientalis.* The ordinate indicates duration nodes of observation.

All the indoor and *in vitro* observation data of the secretion and retraction continuous change processes of unpollinated PDs of the AC, HAC, and TAC were analyzed to obtain the overall patterns of the PDs in each age group (**Figures 3**, **4**). After secretion, the unpollinated PDs in each age group persisted for approximately 3 days before gradual retraction. The duration of AC PDs (79 h 19 min) was significantly longer than that of HAC (60 h 48 min) and TAC PDs (59 h 21 min), and the appearance time of the PDs continued to retreat with increasing tree age. Although the maximum diameters of the PDs at the fullest stage were not significantly different among the age groups (0.392 mm, 0.376 mm and 0.369 mm for the AC, HAC and TAC groups, respectively), the maximum diameters of the PDs decreased with age. In general, the three groups exhibited a matryoshka relationship; at most time points, the diameter of the PDs decreased with increasing tree age. In addition, **Figure 3** shows that the change process of the PDs was similar at each age stage; the PDs all underwent approximately the same fluctuation process and reached their peaks at approximately 23 h, 46 h, and 70 h, separated by approximately 1 day. However, the magnitude of fluctuation in the PDs of the three age groups differed, and the variation gradually increased with increasing tree age, while the variation of the AC PDs was not significant.

**Figure 5:**
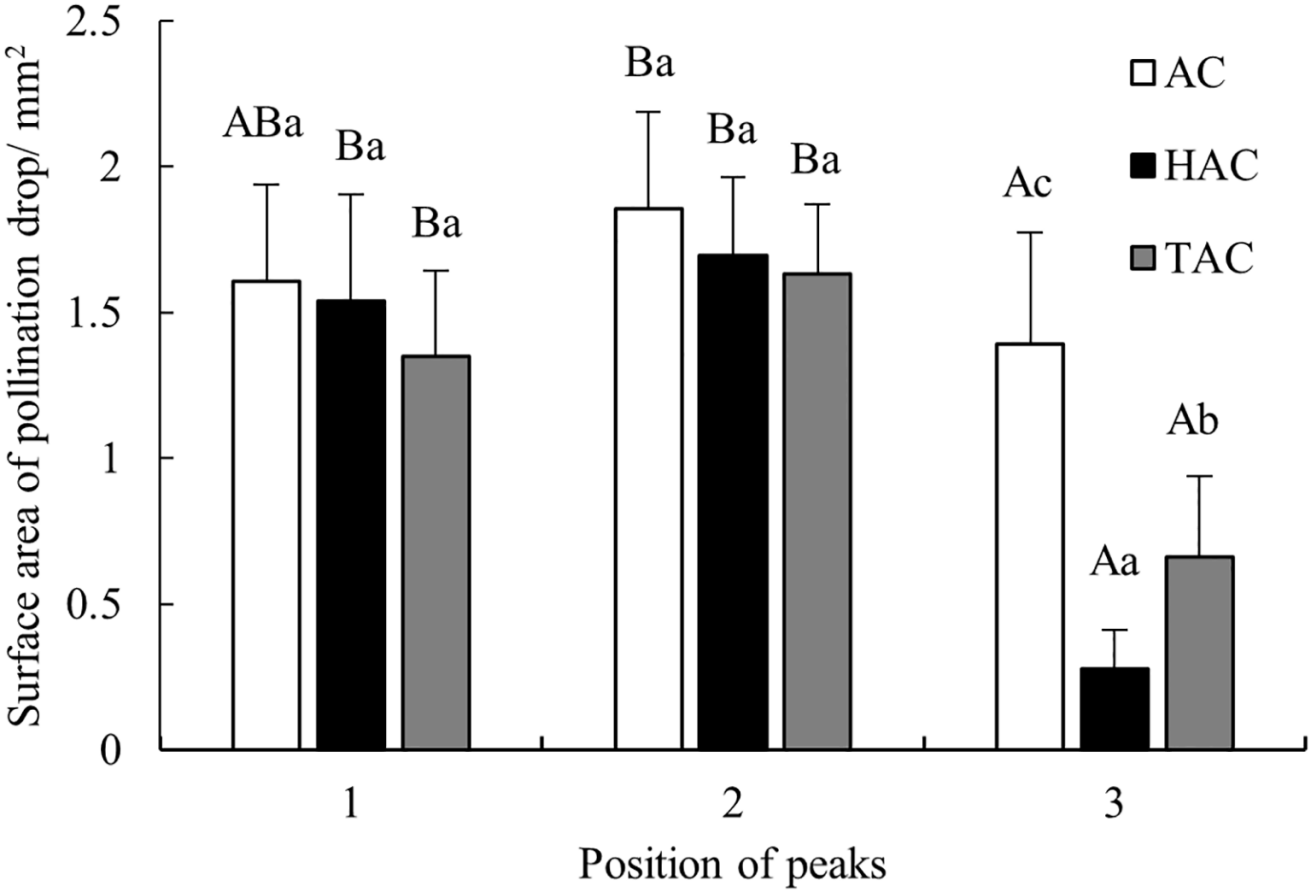
Comparison of peak values of surface area of unpollinated PDs at different ages of *P. orientalis.* Capital letters indicates the significance of differences between different positions of peaks (p < 0.05). Lowercase letters indicates the significance of differences between different ages of *P. orientalis* (p < 0.05).

Surface area is a very important indicator of PDs because the larger the surface area is, the greater the probability that pollen will stick to the PD, and thus the greater the likelihood of successful pollination. Therefore, the horizontal diameter and vertical diameter of each PD in each age group at the three peaks were input into Mathematica to calculate the surface area of each PD, and the mean surface area of the PDs in each age group was calculated (**Figure 5**). The surface area of the unpollinated PDs in each age group changed significantly at the three peak time points, following a consistent pattern. First, the surface area of the PDs at the middle peak was the largest in each age group, i.e., 1.86 mm^2^, 1.69 mm^2^, and 1.63 mm^2^ for the AC, HAC, and TAC groups, respectively. Second, at the first two relatively important peak time points, the peak PD surface area decreased with increasing tree age, which was consistent with the variation in the PD diameter. Finally, the surface area of the PDs at the third peak was the smallest in each age group and was significantly different from those at the previous two peaks (p < 0.05), which may be due to the fact that this PD secretion occurred later and was not complete. Overall, the secretion of unpollinated PDs exhibited rhythmic fluctuations, and there was an overall trend of first increasing and then decreasing.

### 3.2 Responses of *P. orientalis* PDs to pollen with different levels of viability

**Figure 6:**
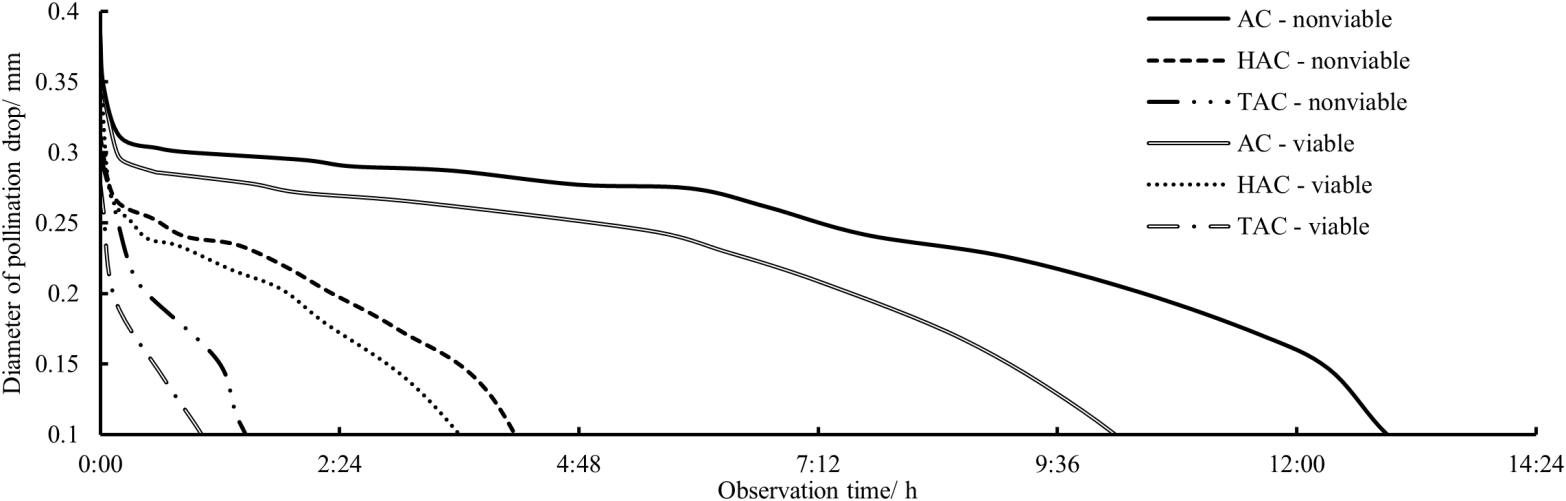
Processes of responses to different pollen viabilities and size changes of PDs at different ages of *P. orientalis.* AC – nonviable represents the PD from AC granted inactivated pollens; HAC – nonviable represents the PD from HAC granted inactivated pollens; TAC – nonviable represents the PD from TAC granted inactivated pollens; AC – viable represents the PD from AC granted normal pollens; HAC – viable represents the PD from HAC granted normal pollens; TAC – viable represents the PD from TAC granted normal pollens.

After the *P. orientalis* PDs in the three age groups were pollinated with viable and non-viable pollen, observations were made of the variation in the continuous retraction of each PD, and the data were analyzed to determine the overall change patterns of PDs in each age group after pollination with pollen of different levels of viability (**Figure 6**). All the PDs were rapidly retracted within a short period after pollination; the retraction slowed rapidly between 1 and 5 min after pollination and continued to gradually slow down from 5–20 min after pollination. However, from 20 min after pollination to full retraction into the micropyle, there was no significant change in the retraction rate, i.e., the retraction rate was nearly constant. Among the age groups, the retraction rate of pollinated PDs was extremely significantly different (p < 0.01), i.e., the higher the tree age, the shorter the retraction time of pollinated PDs, and the average times from pollination to complete retraction of pollinated PDs in the AC, HAC, and TAC groups were 11 h 32 min, 3 h 49 min, and 1 h 8 min, respectively. In addition, for each age group, the overall retraction rate of the PDs pollinated with viable pollen was higher than that of PDs pollinated with inactivated pollen. According to the records of the time to complete retraction, the average time differences of PDs pollinated with the two types of pollen in the AC, HAC, and TAC groups were 2 h 38 min, 53 min and 42 min, respectively; these results are inversely proportional to tree age, but the difference was not significant.

**Figure 7:**
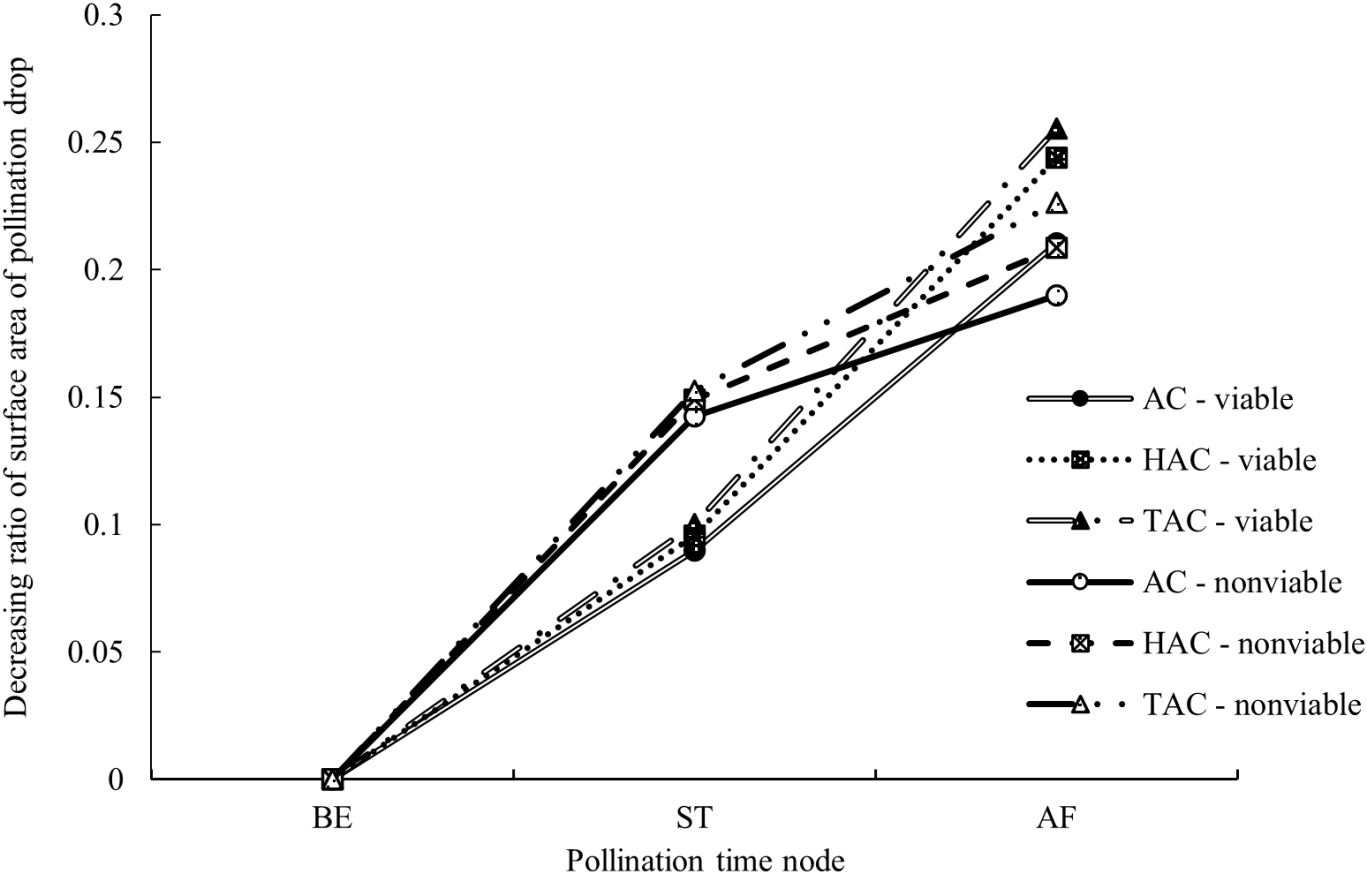
Processes of surface area changes of PDs after pollination at different ages of *P. orientalis.* BE indicates before pollination; ST indicates when pollen is just granted; AF indicates 10 min after pollination. The ordinate indicates the surface area ratio of the difference between the time point and previous time point to BE of the PDs. The legend is same as **Figure 6**.

**Figure 8:**
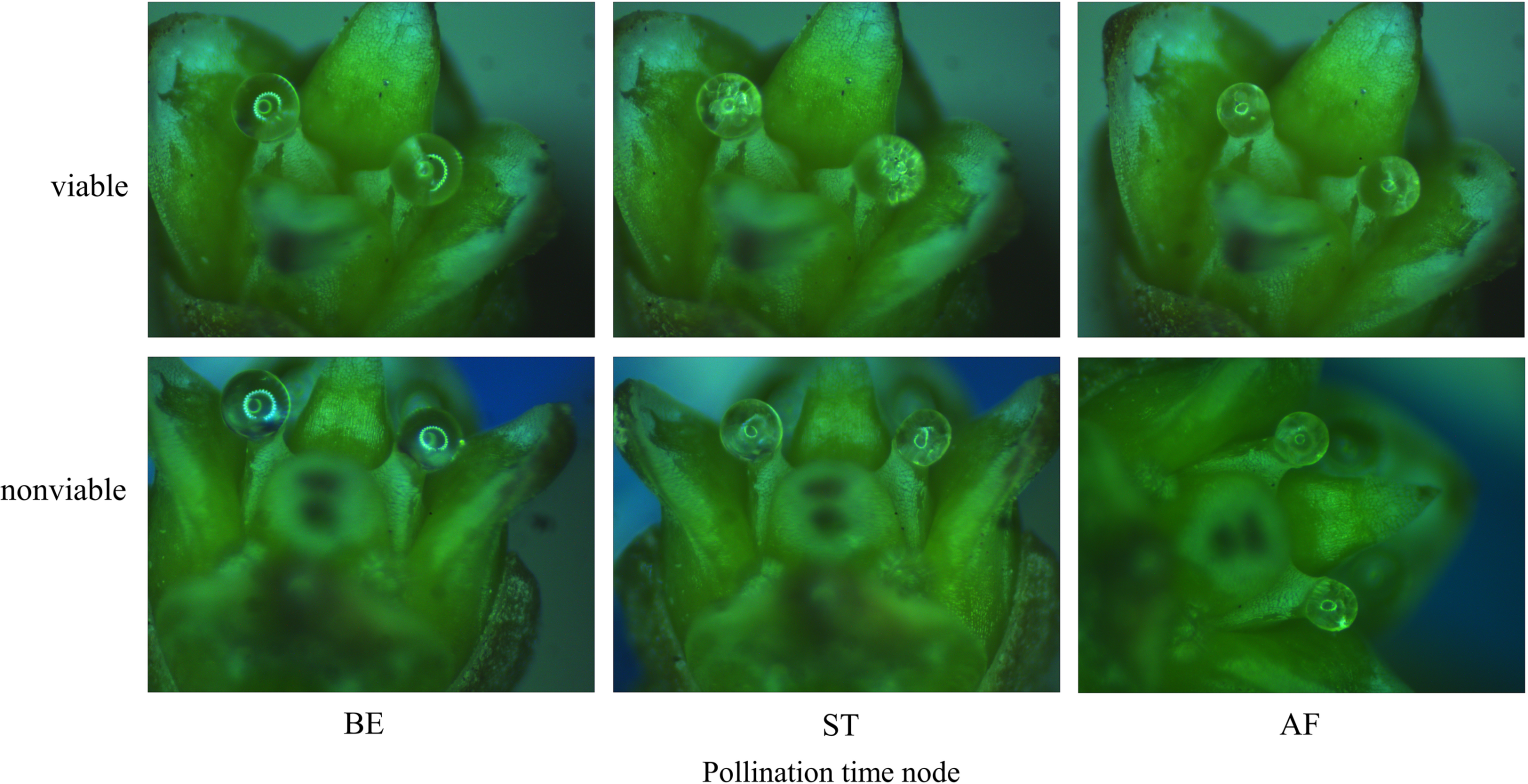
Change processes of PDs of ancient *P. orientalis* after pollination.

Compared with the entire retraction process, changes in PDs occurred very rapidly within a short period immediately after pollination; thus, the changes during this period are not clearly shown in **Figure 6**. The surface area of the PDs in each group was calculated at three time points, i.e., at the time of pollination, 1 min after pollination and 10 min after pollination, and the proportions of the differences between the three time points in the surface area before pollination were calculated to better reflect the changes in the PDs immediately after pollination. As shown in **Figures 7** & **8**, the decrease in and decreasing rate of surface area in each group of PDs after pollination exhibited different trends. The mean proportions of surface area reduction in the PDs of the AC, HAC and TAC that were pollinated with viable pollen were 8.97%, 9.58% and 10.03% within 1 min after pollination, respectively. Similarly, when pollinated with non-viable pollen, the mean proportions of surface area reduction were 14.25%, 14.92% and 15.26%, respectively. The reduction in the surface area of PDs pollinated with non-viable pollen was higher than that of PDs pollinated with viable pollen in all age groups, and the intragroup comparisons of the two pollen treatments showed that the reduction in surface area tended to increase with tree age, but the differences caused by tree age were significantly smaller than those produced by the different pollen types. Between 1 min and 10 min after pollination, the mean proportions of the decrease in the PD surface area of AC, HAC and TAC plants pollinated with viable pollen were 21.03%, 24.38% and 25.54%, respectively. Similarly, when pollinated with non-viable pollen, the mean proportions of the reduction in the PD surface area were 18.99%, 20.86% and 22.64%, respectively. During this period, the patten of the reduction in surface area of PDs pollinated with different pollen types was reversed. In each age group, the reduction in the surface area of PDs pollinated with viable pollen was greater than that of the PDs pollinated with inactivated pollen. The reduction in surface area still tended to increase with tree age, but the trend was more obvious at 1 min after pollination. Compared to those before pollination, the surface areas of the AC, HAC and TAC PDs pollinated with viable pollen ten min after pollination were 70.00%, 66.04% and 64.43%, respectively; similarly, when pollinated with non-viable pollen, the surface areas were 66.765, 64.22% and 62.10%, respectively, compared to those before pollination. These results suggest that the retraction of the PDs pollinated with non-viable pollen in each age group was slightly faster than that of PDs pollinated with viable pollen; however, the PDs pollinated with normal pollen completed retraction before the PDs pollinated with non-viable pollen, and the proportion of retained PD surface area was inversely proportional to tree age.

### 3.3 Responses of *P. orientalis* PDs to pollen from different species

**Figure 9:**
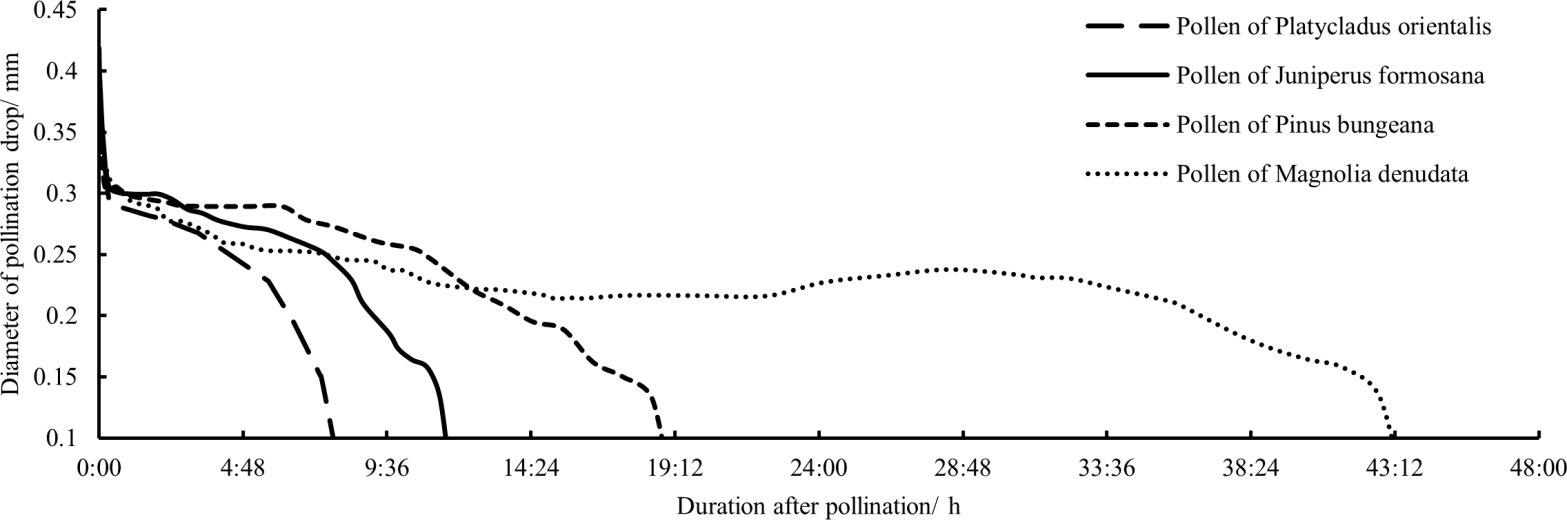
Processes of responses to different species pollens and size changes of PDs in *P. orientalis*.

To understand the differences in the trends of *P. orientalis* PDs pollinated with pollen from different species, in this study, *P. orientalis* PDs of the same size in each age group were pollinated with pollen from *J. formosana*, *P. bungeana*, and *M. denudata*. Observations were made of the post-pollination change in size for each PD, and the data were analyzed to determine the responses of *P. orientalis* PDs in each age group to pollen from different species. **Figure 9** shows the average results of the retraction process of PDs after pollination in each age group. The change process of the PDs pollinated with the gymnosperm pollen was similar to that of the PDs pollinated with *P. orientalis* pollen; for both, the PD was rapidly retracted within a short time after the pollination, followed by a gradual decrease in the retraction rate to varying degrees, and finally the PD was retracted slowly into the micropyle at a nearly constant rate. The only difference was the difference in the time to complete retraction, which gradually increased with the decreasing botanical affinity of the pollen. On average, it took 7 h 29 min, 11 h 42 min and 18 h 56 min to complete retraction after pollination with the pollen of *P. orientalis*, *J. formosana*, and *P. bungeana*, respectively. On the other hand, the PDs pollinated with pollen of *M. denudata* took an average of nearly 2 days to finally retract. There was also a slight increase in the size of the PDs beginning approximately 24 h after pollination, and a small amount of liquid emerged from the fully retracted PDs beginning approximately 52 h after pollination, similar to the process observed for the unpollinated PDs.

For the different tree ages, for both types of pollen, the time for complete retraction of the PDs significantly decreased with increasing age (**Table 1**) (p < 0.05), and the trend was the same for pollen with different levels of viability, indicating that the older the tree was, the higher the retraction rate for the PDs of ancient *P. orientalis*.

**Table 1:** Retraction time of responses to different species pollens of PDs at different ages of *P. orientalis*.

Group	*P. orientalis*	*J. formosana*	*P. bungeana*	*M. denudata*
AC	10 h 11 min (Ac)	19 h 08 min (Bc)	28 h 19 min (Cc)	47 h 59 min (Db)
HAC	3 h 26 min (Ab)	9 h 55 min (Bb)	15 h 42 min (Cb)	39 h 41 min (Da)
TAC	51 min (Aa)	3 h 21 min (Ba)	6 h 57 min (Ca)	35 h 28 min (Da)

Capital letters indicates the significance of differences between different species pollens.

(p < 0.05). Lowercase letters indicates the significance of differences between different ages of *P. orientalis* (p < 0.05).

### 3.4 The secretion and retraction processes of *P. orientalis* PDs in the natural setting

**Figure 10:**
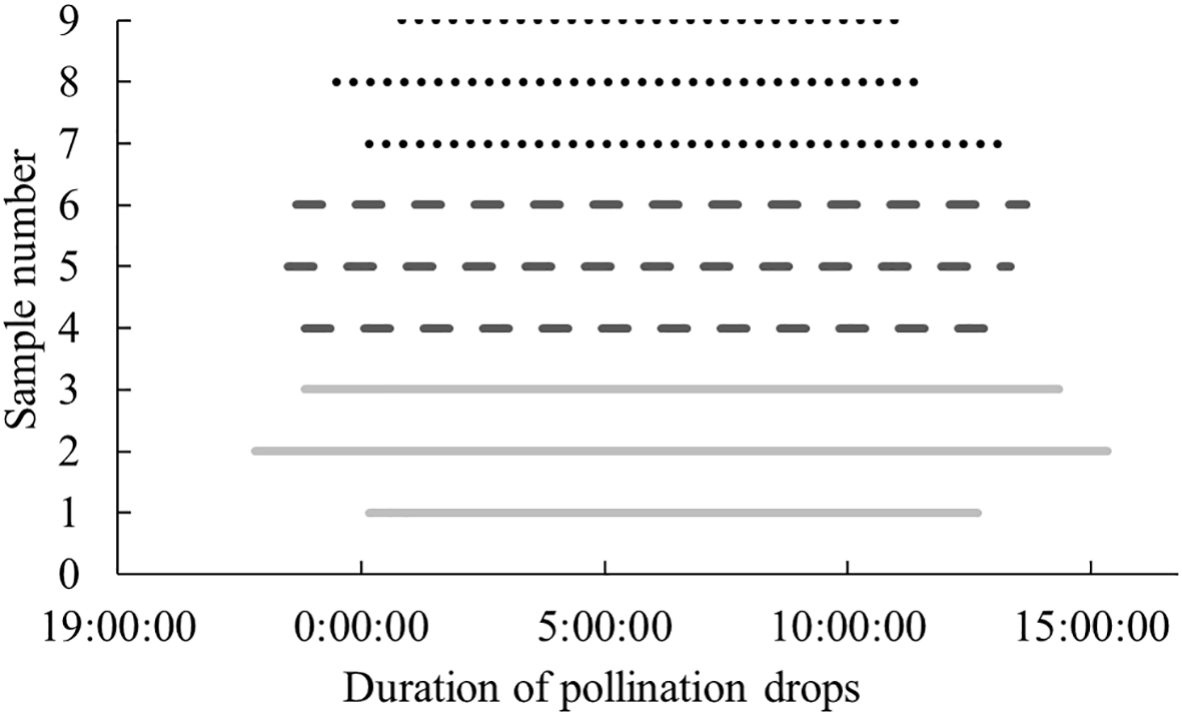
Secreted durations of unpollinated PDs at different ages of *P. orientalis* in natural state. The abscissa shows Beijing time, and the ordinate indicates number of the PDs of different age groups: 1–3 for AC, 4–6 for HAC, and 7–9 for TAC.

**Figure 11:**
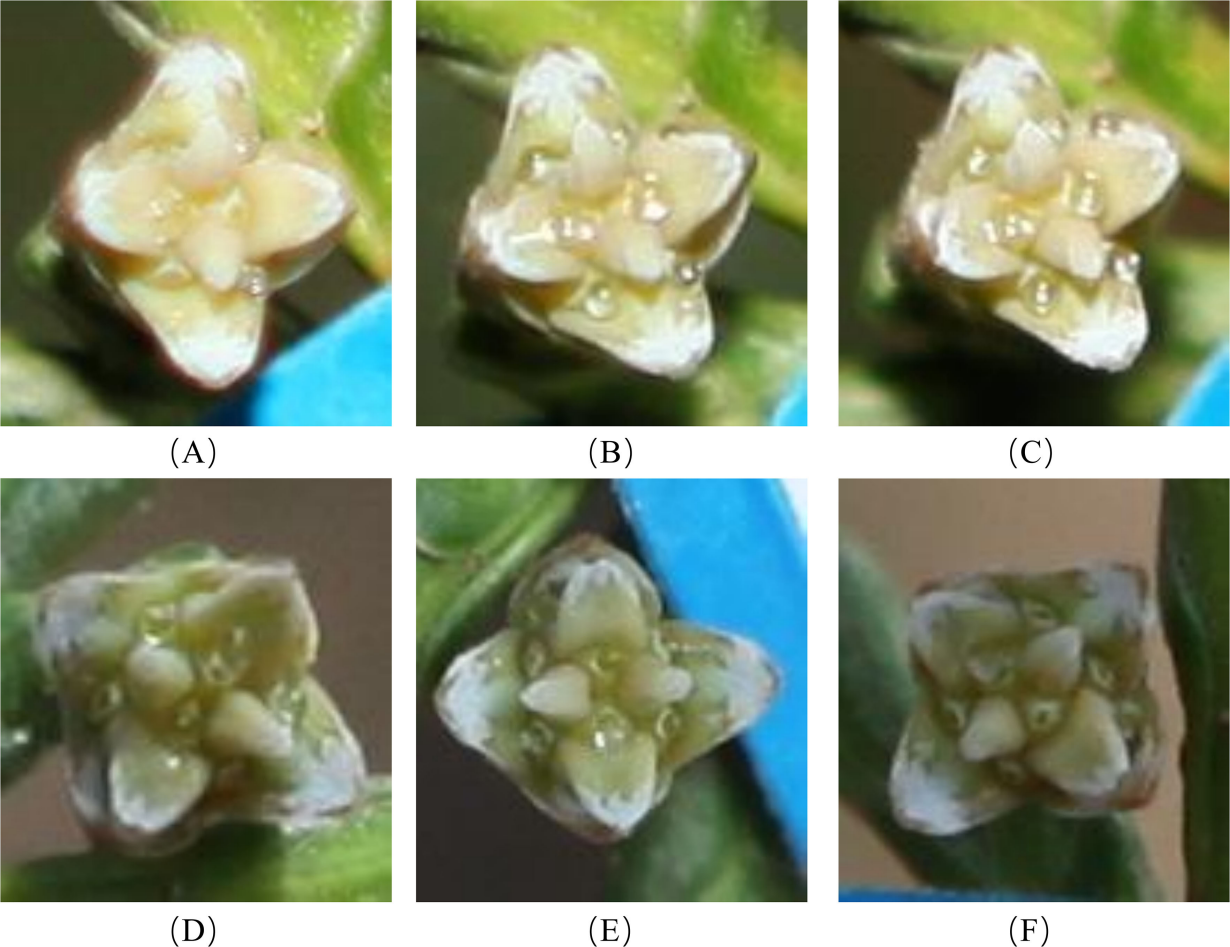
Process of secretion and retraction of unpollinated PDs of ancient *P. orientalis* in natural state of one day. **(A–F)** indicate the states of PDs at 23:00, 2:00, 5:00, 8:00, 11:00, and 14:00, respectively.

Since the conditions of the indoor *in vitro* experiment differed from the natural environment of *P. orientalis*, the results may differ from those observed in the natural environment. To understand the characteristics and patterns of the secretion and retraction processes of *P. orientalis* PDs, the continuous change patterns of PDs before and after pollination were systematically observed and compared with the results of the indoor *in vitro* experiments. As shown in **Figure 10**, in the natural setting, unpollinated PDs in each age group were secreted in the morning and retracted around noon, and the appearance time extended equally to both sides of 6 a.m., which was the midpoint. Moreover, the secretion and retraction cycles were repeated daily, consistent with a circadian rhythm, with a greater cyclic fluctuation than that occurring in the indoor experiments. **Figure 11** shows the states of the *P. orientalis* PDs at different time points throughout the appearance of the PD. A comparison of the durations of PDs among the three age groups showed that the older the tree was, the shorter the duration of the PD. The average durations of the PDs for the AC, HAC and TAC groups were 15 h 10 min, 14 h 36 min and 11 h and 47 min, respectively, so the duration of PDs decreased with increasing tree age. This finding is consistent with the findings of the indoor experiments.

**Figure 12:**
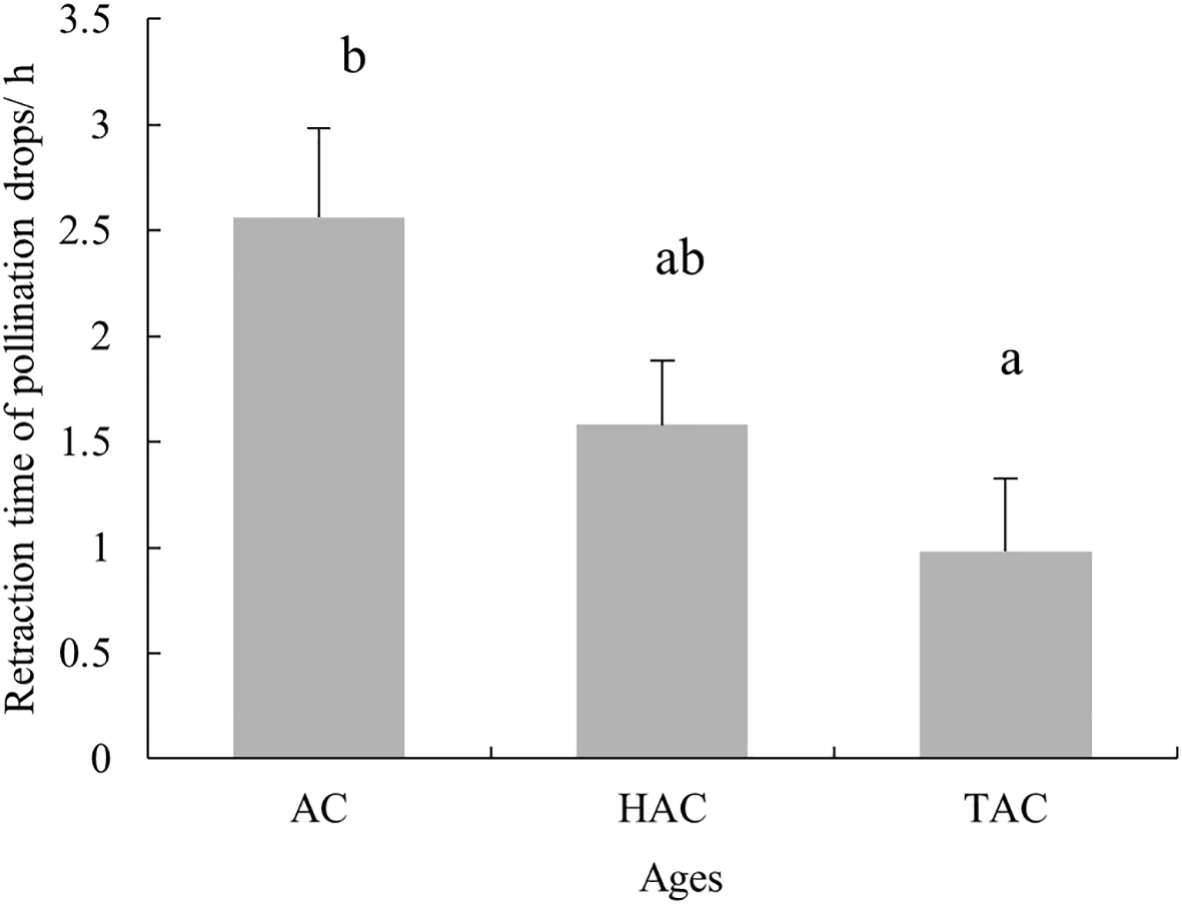
Completion times for retraction of PDs at different ages of *P. orientalis* after pollination in natural state. Lowercase letters indicates the significance of differences between different ages of *P. orientalis* (p < 0.05).

The pollination experiments in the natural setting showed that the retraction time of PDs after pollination decreased with age. The average retraction time of PDs of the HAC was 59 min, which was significantly less than that of the AC (2 h 34 min) (p < 0.05), with that of the TAC falling in between these times (1 h 35 min) (**Figure 12**). These results followed the same trend as that observed for the indoor pollination experiment, but the difference was not as significant as that of the indoor experiment.

## 4 Discussion

PDs are liquid drops secreted by ovules in female gymnosperm cones during the pollination period to increase pollination efficiency. The secretion and retraction phenomena and basic change patterns of various gymnosperm PDs in various states during the pollination period have been reported (Said, 1988; Wang et al., 2009a; Deng et al., 2025), some related research has speculated on the function and mechanism of action of PDs (McWilliam, 1958; Owens et al., 1987; Tomlinson et al., 1997; Anderson and Owens, 1999; Xing et al., 1999; Anderson and Owens, 2000; Jin et al., 2012a, 2012; Dörken and Jagel, 2014; Breygina et al., 2024; Bringas et al., 2025), and various beneficial explorations have been conducted (Owens et al., 1998; Xing et al., 1999; Mugnaini et al., 2007; Little et al., 2014; Muto et al., 2024). However, the relationship between the secretion and retraction patterns of PDs and the age of ancient trees has rarely been reported. In this study, ancient *P. orientalis* tress belonging to two age groups were used to investigate the secretion and retraction patterns of PDs on female cones and the responses of PDs to different pollen types.

In this study, PD diameter was measured in indoor experiments, and the results revealed that the diameter of the normal *P. orientalis* PDs was between 0.2 and 0.5 mm, which is consistent with the findings of Xing et al. (Xing et al., 1999), slightly smaller than that of the genus Taxus (500-600 μm outdoors and 600-800 μm indoors) (Xing et al., 2000a; Wang et al., 2009b), and slightly larger than that of members of the yellow wood family (200-250 μm) (Wang, 2008), suggesting that the PD size does not differ greatly.

### 4.1 Secretion and retraction patterns of nonpollinated *P. orientalis* PDs

The analysis of the secretion and retraction processes of nonpollinated *P. orientalis* PDs in each age group in the indoor and *in vitro* experiments showed that after their appearance, the nonpollinated PDs in each age group persisted for approximately 3 days before they gradually retracted, which is similar to the behavior observed for PDs on isolated branches of different species in indoor hydroponics system (Xing et al., 2000a; Zhang, 2001; Jin et al., 2012a). Regardless of the appearance time or persistence time, the variations in surface area or the circadian rhythm at each time point all showed a gradual weakening trend with age; in particular, the diurnal variation in the secretion and retraction of PDs in the AC group (i.e., the younger trees) was not obvious. Under natural outdoor conditions, the daily persistence time of unpollinated PDs gradually decreased with increasing age, indicating that younger trees had greater secretion power and that the older trees may have a relatively weaker driving force for fluid secretion. The longer the PD lasts and the larger the surface area is, the greater the probability of pollen adhering to it, and thus, the greater the possibility of successful pollination of ovules in female cones. Therefore, the younger trees (those in the AC group) have the advantage of greater competition for reproductive resources, but the difference caused by tree age in this study was not large. Zhao (Zhao, 2009) believed that trees are thought to lose fertility as they age. Wang (Wang, 2016) studied the ancient *Pinus tabuliformis* of Mount Tai and showed that the older the tree was, the more permeable the cell membrane. An increase in cell membrane permeability may cause the water potential of nucellar cells to change, thus affecting the secretion of PDs. The PD secretion rate may also be related to the relaxation time of cellular water exchange (Liu, 2013a). In addition, when PDs are secreted, the cell membranes of the nuclei undulate, and the Golgi apparatus and endoplasmic reticulum surge (Seridi and Chesnoy, 1986; Owens et al., 1987; Seridi-Benkaddour and Chesnoy, 1991; Chesnoy, 1993; Takaso and Owens, 1995), indicating that PD secretion may be related to transmembrane transport. The efficiency of water transmembrane transport in plants is closely related to aquaporins (Chaumont and Tyerman, 2014; Sun et al., 2015; Bao et al., 2017; Wang and Zhao, 2023). Li et al. (Li et al., 2006) cloned GhAQP1, an aquaporin specifically expressed in ovules and regulated by ovule development, and Li (Li, 2010) discovered that from 8 days after anthesis to 21 days after anthesis, the expression level of aquaporin genes in ovules gradually increased. Therefore, the activity and abundance of aquaporins may also affect the secretion efficiency of PDs. A negative correlation between aquaporin activity in nucellar cells and tree age could also explain the relatively small PDs of ancient trees. Several studies have shown that aquaporin activity is regulated by a gating mechanism, and the influencing factors include heterologous genotyping, pH, solute gradient, phosphorylation, Ca^2+^, osmotic pressure, and temperature (Xu et al., 2019); nutrient deficiency, salinity stress, HgCl_2_, hypoxia, and low temperature can also inhibit aquaporin activity (Zhang and Du, 2014; Wu et al., 2015; Jiang et al., 2018; Ding et al., 2024). The influencing factors are very extensive; however, there are no reports on the correlation between the activity or abundance of aquaporins and tree age, and this topic remains to be further explored by scholars. In addition, many studies have shown that nucellar cells disintegrate before PD secretion, that PD secretion is directly related to the degradation of nucellar tissues (Lill and Sweet, 1977; Owens et al., 1980, 1987; Tomlinson et al., 1991; Carafa et al., 1992; Xing et al., 2000a; Muto et al., 2024), and that the degree of degradation may be related to the secretion efficiency of the PDs. However, there are no reports on the effect of tree age on the degradation of nucellar tissue, and further study is needed.

The observation of unpollinated PDs in the natural setting showed that the PDs of each age group were secreted in the early morning and retracted around noon, and the appearance time extended equally to both sides, with 6 a.m. as the midpoint. Moreover, the secretion and retraction cycles were repeated daily, indicating circadian rhythms. This result is similar to the results of many studies on different plants (Baird, 1953; McWilliam, 1958; Owens et al., 1980; Wang et al., 1997; Xing et al., 2000a; Liu, 2013b). Xing et al. (Xing et al., 2000a) suggested that the retraction of *Taxus chinensis* PDs was completed in the morning. However, Wang et al. (Wang et al., 2009b) reported that the PDs of *Taxus yunnanensis* were produced successively for more than three months, from December to March of the next year, with a very long duration, and the PDs were not limited by the pollinating period, i.e., a single PD can last for only between 15 min and 4 h in the pollinating period but for as long as 10 h to 17 d in the nonpollinating period (Wang, 2008). Owens et al. (Owens et al., 1980) suggested that when the ovule of *Chamaecyparis nootkatensis* was not pollinated, it could secret and retract 2–4 PDs, and the duration of each PD was different. Moreover, Owens et al. (Owens et al., 1987) suggested that each ovule of *Picea engelmannii* produced only one PD; in the absence of pollination, the PD can be retained for several days continuously, and its volume can be reduced but does not disappear by noon and can be recovered during the night. Ren (Ren, 1989) reached a similar conclusion in a study on *Torreya grandis*; that is, PDs can appear continuously for at least 5–7 days before drying. For *welwitschia mirabilis*, PDs can appear continuously for approximately 10 days without changes in the circadian rhythm (Carafa et al., 1992). *Pinus radiata* produces PDs only when the air humidity is high (Lill and Sweet, 1977). These unique differences may be associated with differences in plant species and climatic and environmental conditions, such as temperature and humidity, among the different regions.

Ren (Ren, 1989) suggested that the secretion and retraction of PDs are controlled by the temperature of the female cones, and the sensitivity temperature is 15°C; that is, PDs occur when the temperature is higher than 15°C, and PDs are retracted when the temperature is lower than 15°C. Ren believed that this process is not directly related to light or weather. Zhang (Zhang, 2014) supported this point of view but suggested that PDs occur when the average daily temperature is consistently above 14°C, and PDs are retracted when the temperature falls below 11°C. Studies on PD secretion and retraction patterns of *Metasequoia glyptostroboides* have reached similar conclusions (for example, the average daily temperature must be above 5°C for PD secretion), but the critical temperature of different plants varies. Anderson and Owens (Anderson and Owens, 2000) suggested that PD secretion was related only to daily temperature and not to the effective accumulated temperature of the PD. However, these inferences are not consistent with the observations in this study. It is not clear whether this difference is due to tree species or other factors, and further investigation is needed.

In this study, the entire secretion and retraction process of unpollinated PDs was also studied indoors. Although the PDs on female cones of *P. orientalis* twigs of various ages cultured *in vitro* persisted, there were still varying degrees of rhythmic changes in their volume. During the monitoring period, there were three peaks in the change in PD size, with the peaks occurring exactly between 13:00 and 16:00 every afternoon; this result differed from the peak time period observed outdoors (i.e., in the natural setting). This is due to differences in conditions, such as lighting, between the indoor and outdoor settings. However, the change process of PDs of the isolated *P. orientalis* twigs also can be explained by the circadian rhythm. The fluctuation of unpollinated PDs was greater in the natural setting than in the indoor experiment, which may be due to the difference in the adequacy of the water supply. In the indoor experiment, the twigs were inserted into water-laden sponges, and the water supply was much more abundant than that of individuals growing outside in soils on the Loess Plateau. In addition, differences in environmental conditions, such as temperature, humidity, and light, may affect the secretion and duration of PDs.

### 4.2 Study on the retraction mechanism of *P. orientalis* PDs after pollination

The retraction of PDs with pollen is an important step in transporting pollen into ovules; however, this process is complex and involves many physical and physiological processes. Doyle (Doyle, 1935) discovered that the pollinated PDs of the *Pinus* genus retracted within approximately half an hour after pollination, while the adjacent nonpollinated PDs did not retract; this observation led to the first proposal that the PDs of gymnosperms actively retract. At present, uncertainty remains around the retraction mechanism of PDs. Wang et al. (Wang et al., 2015) summarized the mechanism into three models: metabolic retraction, nonmetabolic retraction and active absorption. These mechanisms most likely work together, and different species may have their own dominant factors. In this study, experiments were conducted on *P. orientalis* PDs of three age groups pollinated with different types of pollen under indoor and natural outdoor conditions to investigate the retraction mechanism of PDs and the differences among age groups.

The rapid, short-term retraction of PDs after pollination (**Figure 7**) confirmed the findings of previous studies on the PDs of *T. chinensis* (Xing et al., 2000a; Wang, 2008), and *Cephalotaxus sinensis*, *M. glyptostroboides*, and *Ginkgo biloba* also have similar characteristics (Friedman, 1987; Xing, 2000b; Lu et al., 2009). It is generally believed that such retraction is caused mainly by the significant absorption of water by the PDs or the change in the surface tension of the PDs following contact with dry pollen (Owens et al., 1980; Gelbart and von Aderkas, 2002). Within a short period after pollination, mutual information exchange between PDs and pollen has not yet occurred, and ovules do not initiate active adsorption quickly. Therefore, the reduction in PDs during this period should be caused by various physical effects of dry pollen; this finding is consistent with the evaporation equilibrium theory, i.e., the non-metabolic retraction process proposed by Xing et al. (Xing et al., 1999) and Jin et al. (Jin et al., 2012b), which is partially supported by some scholars (Owens et al., 1987; Carafa et al., 1992). At 1 min after pollination, the differences in the absorption of different types of active pollen by PDs were relatively large, and the absorption of inactivated pollen was greater. Therefore, the water absorption and storage properties of inactivated pollen may be better than those of viable pollen, and the differences in retraction due to differences in pollen viability also supported the effect of pollen on adsorption by PDs. However, between 1 and 10 min after pollination, the intergroup difference in the response of PDs to viable pollen was reversed, the decrease in the surface area of PDs pollinated with viable pollen was greater, and the intragroup differences among the tree ages increased. These findings indicate that non-metabolic water adsorption by pollen and active adsorption by ovules may act together at this stage, but the effect gradually weakens after pollen adsorption reaches saturation.

However, the pollen adsorption theory cannot effectively explain the continuous retraction of *P. orientalis* PDs over several hours. This may be caused by the active retraction effect dominated by recognition. At 10 min after pollination, the number of remaining PDs was higher among those pollinated with viable pollen than among those pollinated with inactivated pollen; however, the retraction of PDs pollinated with viable pollen was completed earlier than that of PDs pollinated with inactivated pollen, indicating that the affinity and identification/selection between PDs and pollen play a role in this process, accelerating the active adsorption of viable pollen by ovules. Some scholars hold a similar view (Coulter et al., 2012; Jin et al., 2012b; Liu, 2013b; Wang et al., 2015). Experiments on the responses of PDs to pollen with different levels of viability and from different species showed that the retraction rate of *P. orientalis* PDs decreased with decreasing pollen viability and decreased significantly with decreasing botanical affinity. This finding is consistent with the conclusion of Xing et al. (Xing et al., 1999). Many scholars have obtained similar results when studying the PDs of different species (Jin et al., 2012b; Dörken and Jagel, 2014; Jin et al., 2020; Deng et al., 2025). These results suggest that there may be substances in PDs that identify pollen and that there may be specific forms of mutual identification or information exchange and conduction with pollen. Moreover, the identification of PDs may involve more than one substance or mechanism any may be the result of comprehensive effects. This comprehensive effect is closely related to the genetic distance of the pollen, which gradually decreases as the botanical affinity of the pollen decreases. Information about the degree of matching that is obtained through the identification process is subsequently transmitted to the ovule tissue to regulate the format and rate of active adsorption.

However, Tomlinson et al. (Tomlinson et al., 1997) suggested that the PDs of *Phyllocladus* do not discriminate against the pollen of different species. Therefore, the mechanisms of PD retraction vary among plant species, which may be the result of long-term evolution of plants in their natural habitats, and additional in-depth systematic studies are needed to further understand these points.

### 4.3 Differences in the retraction of *P. orientalis* PDs after pollination

Since the number of pollen grains used for each pollination effort was roughly the same in this study, the number of PDs needed to consume the pollen originating from the same species should also be roughly equal. The change in the surface area of PDs immediately after pollination was studied (**Figure 7**), and the results showed that at 1 min after pollination, although the reduction in the surface area of PDs that received pollen from the same species was basically the same, there was a slight increasing trend with tree age. This finding may be attributed to the fact that when PDs are pollinated, their emergency response mechanism is triggered. PDs of each age group may have different degrees of interaction with pollen immediately after pollination. However, since the difference in the change in surface area between PDs of each age group was very small, this effect may be very weak.

The retraction rate of *P. orientalis* PDs in each age group showed that the older the tree was, the faster the retraction. The average times for complete retraction of the PDs of the AC, HAC and TAC in the natural setting were 2 h 34 min, 1 h 35 min and 59 min, and those of PDs in the isolated culture setting were 11 h 32 min, 3 h 49 min and 1 h 8 min, respectively, indicating that the PDs of trees of all ages retracted faster in the natural setting, which is similar to the results of the changes in unpollinated PDs and may be related to the abundance of the water supply. Xing et al. (Xing et al., 1999), Dörken and Jagel (Dörken and Jagel, 2014) reported that *P. orientalis* PDs can be completely retracted within 100 min and 20–30 min after pollination. The existence of such significant differences within the same plant may be caused by differences in environmental factors or experimental conditions. There are also large differences in the retraction rates of PDs among different species, and PDs of Engelmann spruce (Owens et al., 1987), *Phyllocladus* (Tomlinson et al., 1997), *Juniperus chinensis* (Liu, 2013b), Cupressaceae (Dörken and Jagel, 2014), pacific yew (Anderson and Owens, 1999, 2000), European yew (*Taxus baccata*) (Dörken and Jagel, 2014), *T. yunnanensis* (Wang, 2008), *Cunninghamia lanceolata* (Deng et al., 2025) and *G. biloba* (Jiang, 2019) can fully retract within 10 min, 4–20 min, 15–20 min, 8–24 min, 30 min, 45–65 min, 45–65 min, 20 min-2 h, 0.46 ± 0.06 h - 3.43 ± 0.84 h and 4 h, respectively; PDs of *T. chinensis* and *C. sinensis* completely retract 20–24 h after artificial pollination (Xing et al., 2000a; Xing, 2000b), and PDs of *M. glyptostroboides* can dry up on the second day after pollination.

Under normal circumstances, the faster the PD retracts, the less likely it is that the retraction process could be affected by external forces, such as the environment, animals, plants, and pathogens, the less likely it is that other factors could affect the pollination process, and the greater the pollination success rate, i.e., the stronger the reproductive ability. However, this study showed that regardless of whether the experiment was conducted in a natural setting or in an *in vitro* culture setting, the PDs produced by older *P. orientalis* trees took a significantly shorter time to completely retract after pollination, indicating that the female fecundity of ancient *P. orientalis* trees gradually increases with tree age, with ancient trees being more inclined to develop female fecundity. The reason for this pattern may be that the older the tree is, the greater the priority female cones are and the more efficient the use and allocation of resources; as a result, a more rapid and violent response occurs throughout the retraction process, and the driving force is also stronger. This speculation relies on further verification of genetic technology.

Altogether, the PD secretion results showed that PD secretion and adsorption may be two mutually exclusive processes that cannot be considered at the same time. In this study, the younger the tree was, the stronger the driving force of secretion was, as demonstrated by the faster secretion, greater volume, and longer duration of PDs; moreover, the older the tree was, the stronger the secretion driving force, as manifested by the significantly higher retraction rate of PDs after pollination. The stronger the secretion was, the greater the relative probability of capturing pollen; the stronger the retraction was, the lower the variability in the retraction process; each process has its own advantages and disadvantages. In this study, the decrease in the driving force of secretion was significantly weaker than the increase in the driving force of absorption, which also indicates that the older the tree was, the stronger the female reproductive ability of ancient *P. orientalis* trees.

Further analysis revealed that the ratios of the total retraction time of PDs in the same age group that were pollinated with two kinds of pollen (viable and non-viable) to the average PD retraction time could characterize the difference in the responses of PDs to the two kinds of pollen. These ratios were 22.8%, 23.2% and 61.8% for the AC, HAC and TAC, respectively, and the ratio gradually increased with tree age. The ratio of the TAC was significantly larger than that of the HAC and AC, indicating that the older the tree was, the more drastic the difference in the response of the PDs to different types of pollen. Similar results were found when PDs were pollinated with pollen from different species, suggesting that the ability of PDs of ancient *P. orientalis* to identify and respond to pollen gradually increased with tree age.

In summary, an analysis of the variation in PDs of ancient *P. orientalis* showed that the reproductive ability of female cones gradually increased with tree age.

## 5 Conclusion

PDs are the secretions produced by the ovules of gymnosperms during pollination and greatly improve the pollination efficiency of gymnosperms. In this study, which focused on the PDs of ancient *P. orientalis*, the secretion and retraction processes of PDs in each age group under natural and *in vitro* conditions were quantitatively analyzed, and the responses of PDs in each age group to pollen with varying levels of viability and from different species were investigated. The results showed that, under natural conditions, the daily duration of nonpollinated PDs decreased with tree age; the secretion time, persistence time, surface area at each time point and rhythmic changes in unpollinated PDs under *in vitro* conditions all weakened with tree age, indicating that the younger the tree was, the more powerful the secretion of PDs. Under natural conditions, PDs were secreted in the early morning and were retracted around noon. The appearance time extended equally to both sides of 6 a.m., which was the midpoint, and the secretion and retraction processes followed a daily circadian rhythm in. The PDs were retracted rapidly right after pollination, the retraction rate gradually slowed for the next 20 min, and the retraction rate remained almost constant thereafter. After pollination, the retraction rate of PDs decreased with decreasing pollen viability and significantly decreased with decreasing botanical affinity. These results suggest that substances that play a role in the identification of pollen and related mechanisms may exist in PDs but that these substances are unrelated to the existence of pollen. This finding suggests the presence of substances related to pollen identification in PDs, a combined mechanism of action at different stages, and specific forms of identification or communication with pollen. The water absorption and storage properties of inactivated pollen may be better than those of viable pollen, and PDs may interact with pollen for a short period after pollination. In the natural setting, PDs were retracted at a faster rate, and the older the tree was, the shorter the time needed for complete retraction of PDs after pollination. The secretion and absorption of PDs may be two mutually exclusive functions that cannot be considered together. The ability of PDs of ancient *P. orientalis* to identify and respond to pollen increases with age, and the older the tree is, the stronger the reproductive ability of female cones.

## 6 Further research

The above conclusions are mainly based on the observations and analysis of biological experimental phenomena. Further verification of these underlying mechanisms requires advances in microscopic research. It is necessary to conduct further research firstly on the components and physiological indicators of the PD to understand the more comprehensive functions of each component, especially hormones and proteins, and determine whether there is recognition or synergistic interaction with conspecific or closely related pollen. Then, it is necessary to investigate and characterize the proteome and metabolome of the PD and pollen, and even explore the genome and transcriptome of ovules and pollen, with particular attention to aquaporins. In this way is it possible to clarify the secretion and retraction mechanisms of the PD at a deeper level, as well as the reasons for their diverse manifestations with tree age.

## Data Availability

The raw data supporting the conclusions of this article will be made available by the authors, without undue reservation.
